# Revisiting the Instrumented Romberg Test: Can Today’s Technology Offer a Risk-of-Fall Screening Device for Senior Citizens? An Experience-Based Approach

**DOI:** 10.3390/life11020161

**Published:** 2021-02-20

**Authors:** Michele Gallamini, Giorgio Piastra, Simonetta Lucarini, Debora Porzio, Matteo Ronchi, Alessio Pirino, Fabio Scoppa, Stefano Masiero, Lucrezia Tognolo

**Affiliations:** 1 Eng. Freelance MD Consultant, Sal. Maggiolo di Nervi, 16167 Genoa, Italy; michelegallamini@gmail.com; 2 Ben-Essere Sport and Wellness Association Rapallo, Third-Sector Liguria Region Registry, 16135 Genoa, Italy; debora.p@ben-essere-asd.it (D.P.); matteo.r@ben-essere-asd.it (M.R.); 3 ASL 4 Liguria (Liguria Regional Health Service), Sports Medicine, N.S. di Montallegro Hospital, 16035 Rapallo, Italy; gpiastra@asl4.liguria.it; 4 ASL 4 Liguria (Liguria Regional Health Service), Geriatric Service, Chiavari, 16043 Chiavari, Italy; slucarini@asl4.liguria.it; 5 Department of Biomedical Sciences, University of Sassari, 07100 Sassari, Italy; axelpir@uniss.it; 6 Faculty of Medicine and Dental Surgery, Sapienza University of Rome, 00185 Rome, Italy; fabioscoppa@chinesis.org; 7 Chinesis I.F.O.P. Istituto di Formazione in Osteopatia e in Posturologia, Osteopathy School, 00152 Rome, Italy; 8 Department of Neurosciences, Physical Medicine and Rehabilitation School, University of Padua, 35128 Padua, Italy; stef.masiero@unipd.it; 9 Rehabilitation Unit, Laboratory of Robotic and Bioengineering and Clinical of Movement, Padua University-General Hospital, 35128 Padua, Italy

**Keywords:** risk-of-fall, balance, postural control, unperturbed upright stance, force plate, Romberg test

## Abstract

Risk of fall (ROF) is a worldwide major concern for its prevalence and consequent dramatic outcomes in the elderly population. The growing age-related risk appears to be associated with increasing motor, sensory, and cognitive problems in the elderly population. There is a consensus on the need to screen for these balance dysfunctions, but the available methods are largely based on subjectively assessed performances. The instrumented Romberg test using a force plate represents a validated assessment process for the evaluation of balance performances. The purpose of this study is to propose an innovative instrumental method to identify balance deficits, assess their severity, and give an automated indication of the most likely etiology. The proposed new method was applied to the instrumented Romberg test, using force plate data recorded in a cohort of 551 females aged >65 participating in adapted physical activity courses. The method allowed us to identify 145 dysfunctional subjects and to determine the likely origin of their deficit: 21 central, 5 vestibular, 9 visual, 59 proprioceptive (musculoskeletal etiology), and 51 functional. Based on the preliminary findings of the study, this test could be an efficient and cost-effective mass screening tool for identifying subjects at risk of fall, since the procedure proves to be rapid, non-invasive, and apparently devoid of any contraindications.

## 1. Introduction

Risk of Fall (ROF) is a worldwide problem due to its social and healthcare implications. In the European Countries (EC), balance deficits are an ongoing health concern, and the main risk factor for potentially fatal falls in the elderly population. Data published in 2015 by the Prevention of Falls Network for Dissemination initiative (ProFouND) and the EuroSafe injury prevention association [1] clearly shows the gravity of the issue: out of a population of 100 Mn individuals, 36,000 fatal injuries due to falls were recorded that year, and 2.3 Mn individuals were consequently hospitalized. Fall prevention is and has been one of the EC priorities in various funding programs, including the Horizon 2020.

The statistics published by the United States Centers for Disease Control and Prevention [2,3] are not different, making it a growing concern for U.S. health services [4]. In Hong Kong, a 2005 study reported that around 27% fallers were aged over 70 [5].

Falls are generally multifactorial in origin [6,7], and it is generally agreed [8] that the best course of action involves identifying persons at ROF, followed by the most appropriate diagnostic path to identify the origin of the deficit. The most predictive factors for future falls [9,10] are the presence of motor problems (gait or balance impairment; muscle weakness), sensory impairment (peripheral neuropathy, vestibular dysfunction, vision impairment) [11,12,13,14], cognitive or mood impairment (dementia, depression, delirium), orthostatic hypotension [15], polypharmacy or certain medicines (particularly psychotropic medicines) [16], and impairment of activities of daily living, environmental hazards (e.g., loose rugs, poor lighting, clutter), as well as additional factors, such as age or comorbidities [17,18,19,20].

There is a consensus among geriatrists that motor problems and sensory impairment should be added to the classical conditions, which constitute a diagnosis of “frailty”, defined as “a clinical syndrome in which three or more of the following criteria are present: unintentional weight loss, self-reported exhaustion, weakness, slow walking speed, and low physical activity” [21].

A survey of the epidemiology [22] of falls shows a rise in fall incidence with age, and an age threshold of around 65 is widely accepted. In 2019, the Task Force of the International Conference of Frailty and Sarcopenia Research (ICFSR) issued recommendations for the identification and management of frailty in older adults, proposing that “all adults aged 65 years and over should be offered opportunistic screening for frailty using a simple, validated frailty instrument suitable to the specific setting or context” [23].

Keeping an upright stance is no easy task [24]: our center of mass (CoM) lies over the soles of the feet and slightly forward of the ankle axis, while an unbalancing torque makes the body prone to stumbling forwards. By exerting a pressure on the ground through the soles of the feet, we create a balancing torque that prevents us from falling: this pressure is applied to a point known as the center of pressure (CoP), the path of which can be analyzed during the test to identify clinically significant indications.

**Figure 1:**
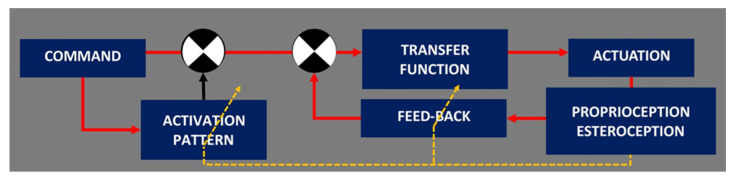
The cybernetic model of motor activities. The model shows the combination of feed-forward (Activation Pattern), of feed-back, and the adaptive modifications of both functions.

Postural control is based on an adaptive mechanism, one which is influenced by a variety of factors, including growth phase, physical or mental health deterioration, diseases, and painful conditions, and which involves both feed-forward and feed-back circuits to continuously update the body’s functional responses [25,26,27,28,29,30,31] (Figure 1).

The Romberg test [32,33] detects the presence of proprioceptive deficits [34]: by comparing closed and open eyes results, specific anomalies were found to be associated with sway control deficits [35] of central [36,37,38,39,40,41,42], vestibular [43,44,45], visual [46], musculoskeletal [47,48,49], and pain-related [50,51] etiology. Sensor and processing technology currently allow balance (and indirectly ROF) assessment using the following instruments: gait analysis; baropodometric analysis; static platforms; mobile platform tests; accelerometric segment motion detectors. However, these techniques present different limitations, making them not fully appropriate for the Romberg test.

The wide variability of the classical parameters does not permit a clear distinction between functional and dysfunctional individuals [52]. To date, balance assessment has largely been dependent on the subjective evaluation of a wide array of parameters, requiring the final diagnosis to be made by highly specialized MDs, making it unsuitable for use as a mass screening tool.

The recent introduction of the “score of postural functionality” (SPF) parameter [53] has eradicated some of the difficulties of performing a reliable diagnostic assessment based on traditional parameters, suggesting the most likely etiology of the observed dysfunction and allowing the most appropriate specialist diagnosis and treatment options to be identified. The definition of a Score of Postural Functionality (SPF), a process discussed at length in a previous paper, offers an apparently affordable approach. 

SPF is the count of anomalies in the 27 Sway Parameters: i.e., parameters with a value outside the range of +/−2 std dev from the normal value. To them, there were added also 36 ratios between the above parameters with a correlation greater than 70% to the mean parameters. The idea is that such ratios contain some “hidden information” about the individual control strategy. This is the heuristic basis for SPF proposal.

**Figure 2:**
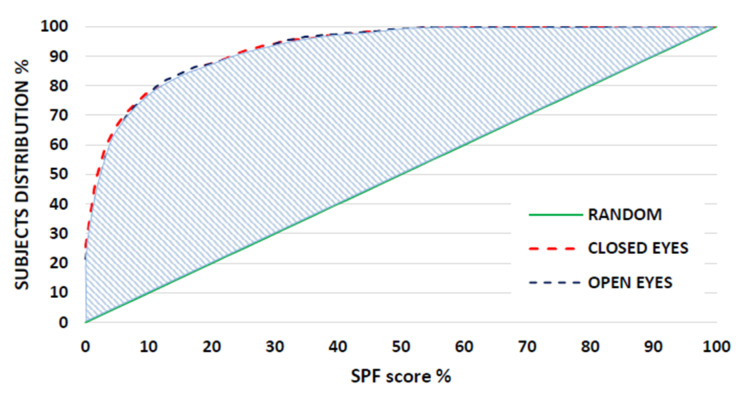
Score of Postural Functionality (SPF) and receiver operating characteristics (ROC). The Area Under the Curve (AUC) is by far over the one that affords 50% of Sensitivity and Specificity, confirming the diagnostic validity of SPF.

To validate the process, we then analyzed SPF value distribution in a population of 1626 senior citizens aged prevalently over 65 years of age. According to ROC (receiver operating characteristics) theory [54,55], the SPF sensitivity/specificity graph clearly showed a fairly high score, measured by the AUC (area under the curve), which was significantly higher than the minimal 50% score attained by a non-discriminating (random) test, given that the maximum SPF value applied to 100% dysfunctional subjects and to 0% fully functional subjects. The closest point to the highest sensitivity and specificity is achieved with SPF = 9 ÷ 10, which therefore represents the threshold between functional and dysfunctional (Figure 2). ROC Approach for SPF: the area under the curve (AUC) confirms its diagnostic relevance.

The present paper proposes a new version of the well-known Romberg test, performed on a force plate in order to identify subjects with balance deficits but apparently good functional status, while providing information on the likely type of deficit.

Therefore, the aim of this study was twofold:

1. clinical: to verify whether the test could accelerate the diagnostic process by identifying those individuals with balance deficits in an apparently healthy population, as well as identifying the type of deficit (proprioceptive, vestibular, visual etc.) potentially affecting balance; and
2. methodological: to assess the feasibility of a mass screening test.

## 2. Materials and Methods

### 2.1. Participants

Between February and April 2018, we conducted an observational study on a cohort of senior citizens taking part in an adapted physical activity program (APA) [56].

The participants were 551 female subjects with a median age of 72 years, a median height of 157 cm, a median weight of 64 kg, and a median body mass index (BMI) of 26.02 (Table 1).

Given that the Romberg is a functional test, all female subjects over 65 and able to stand unassisted in an unperturbed stance were included in the study. 

The cohort was extracted from a population of 911 subjects recruited for the test. The selection was limited to:

1. individuals aged over 65, in keeping with the target population for the screening program; and
2. female subjects (almost 80% of the selected population) to give a homogeneous cohort.

The Romberg test has no contraindications and can be applied to every individual capable on keeping the upright unperturbed stance. The participants to the test, actively participating to APA courses, were all fit.

It is worth noting that similar results were observed in the original study population and in the extracted cohort. 

All participants provided written informed consent in accordance with the Declaration of Helsinki.

### 2.2. Test Protocol: Test Execution and Parameters

The static force plate, which measures force components along three axes (X, Y, Z) or a single axis (Z), is an appropriate device for performing the instrumented Romberg test. 

The design is simple: a rigid plate is supported by strain gauge sensors at its corners (three or four sensors), and the vertical component of the ground reaction force (GRF) applied at the CoP is shared between the strain sensors at a ratio which is inversely proportional to the distance of the CoP from each sensor. The CoP position is then easily calculated from the GFR components measured by the device. The process is repeated at constant time intervals according to the selected sample frequency. The CoP path is then expressed as a series of subsequent positions. Using CoP path signal processing, the balance parameters are then calculated and extracted by the clinician for diagnostic assessment. Although most of the parameters provided by the force plate data processing are well known to specialists [57], a summary of them is given in Table 2.

As said, the device is a rather simple one, but several requirements [58,59,60] should be complied with:

1. Its geometry (the vertical distance of the measurement plate from the ground should be as far as possible specular to the distance between ankle axis and foot soles).
2. Its stiffness and inertia should afford a natural oscillation frequency well above the range of expected COP path dynamics.

Analog measurements should be filtered and sampled at a frequency high enough to detect all the harmonics of the body sway (generally under 10 Hz), at a resolution consistent with a dynamic accuracy greater than 0.1 mm throughout the 0–10 Hz band. The sway parameters are then calculated from the CoP path.

The instrumented Romberg test was performed using the ArgoplusMK1 Force Plate^®^ (Fremslife Srl, Genoa, Italy). Table 3 shows the technical characteristics of the force plate.

ArgoPlusMK1^®^ features an accuracy better than 0.1 mm in the COP coordinates measurement up to 10 Hz COP dynamics—the device is CE certified as a measuring instrument—,and its performances have been validated through specific procedures [61]. 

In addition to the classical parameters, ArgoPlusMK1 provides a reliable harmonic analysis [62] (amplitude of X, Y harmonics in the calculated oscillation spectrum); sway density [63] (the structure of the sway resulting from small high-frequency ballistic impulses [64] over low frequency feed-back adjustments); a series of comprehensive parameters (such as the ellipse axis ratio to demonstrate the presence of a preferential oscillation plane, postural tone [65], and the motor control performance index given by the sway density); and the aforementioned SPF measuring the number of anomalies compared to a healthy sample.

The subjects were asked to maintain an upright barefoot (light stockings/socks) standing position on the force plate with feet together and parallel arms hanging loosely at their sides [66,67], mouth closed (with jaw relaxed not clenched), and head up. They were asked to hold this position with closed eyes (CE) for 45 s and then to maintain the same position for 45 s with open eyes (OE) [68]. The tests were conducted by the APA instructors in the same centers where the APA courses were normally held, and as part of the normal course schedule. The environmental conditions were consistent with the mentioned standards defined by Kapteyn et al.

### 2.3. Data Analysis

While it may be tempting to equate a higher SPF with a higher ROF, the SPF does not give the whole picture. While the SPF is certainly helpful in calculating ROF, there is no mathematical correlation between the two variables; indeed, the relationship between the two is not yet entirely clear. 

Balance keeping is based on a complex interplay of biomechanics, sensory feedback and movement patterns which adaptively develop in each individual, meaning that there is likely to be a wide variety of control characteristics. The biomechanical challenge of maintaining balance in an upright stance affords infinite solutions, where the individual “selects” different control features matching their own specific characteristics, and only the cluster approach seems capable of assessing both the effectiveness and the efficiency of balance keeping.

**Scheme 1:**
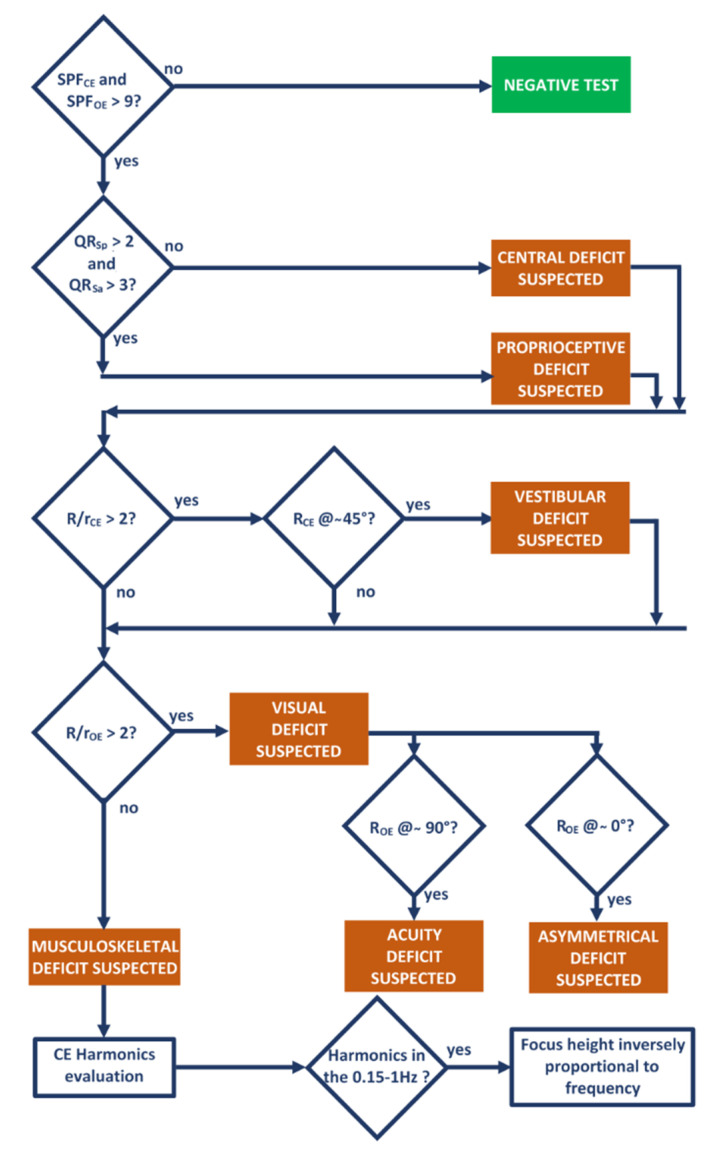
Test assessment flow chart. OE = Open Eyes Test, CE = Closed Eyes Test. SPF = Score of Postural Functionality, QR = Romberg’s Ratio, Sp = Sway Path, Sa = Sway Area, R = Ellipse Major Axis, r = Ellipse Minor Axis.

Based on the above, a potential new approach for the analysis of these parameters is shown in the graph of Scheme 1, where the initial step in the process consists of deciding whether or not the subject is normofunctional, based on the SPF value.

If at least one of the two SPF values is higher than 9, further investigation is recommended to identify the most likely causes of dysfunction.

The second step is analysis of the Romberg quotients (RQ): if the deficit shows no marked difference between the closed and open eyes tests, there may be grounds to suspect a central or structural musculoskeletal deficit. 

When the ellipse shows a marked asymmetry (ratio between axis > 2), there is a preferential oscillation trend: if it is observed mainly in the closed eyes test, and the main axis is lying on a quadrantal direction, a vestibular deficit may be suspected [69].

When the asymmetry is mainly present in the open eyes test, a visual deficit may be suspected, and hints for its interpretation can be taken from the direction of the main axis: a latero-lateral direction suggests a possible asymmetry between the two eyes, while an antero-posterior direction may suggest a visual acuity deficit. This deduction is not yet supported by specific experimental evidence, but a series of episodic experiences gathered from systematic tests of elderly individuals suggests that this hypothesis, based mainly on [46], appears more than reasonable. 

Lastly, the deficit may be of musculoskeletal etiology, and spectral analysis may be helpful for diagnostic purposes.

Based on the inverted pendulum model [70,71], it seems reasonable to assume that the undampened free oscillation harmonics displayed in the unperturbed upright stance will reflect the height of the perturbing factor (the lower the frequency of oscillation, the greater the distance from the ankle axis), with movements ranging from around 0.1 Hz (corresponding to the upper cervical vertebrae) to 1 Hz (corresponding to the ankle axis), a correlation confirmed by experience to a certain extent. 

If the peak harmonic is shown on the sagittal plane, the perturbing factor is either bilateral, or more probably on the sagittal plane; if it is shown on the frontal plane, the perturbing factor is probably to be found on one side of the body (frequencies higher than 0.3 Hz are most likely related to a lower limb impairment or compensation).

### 2.4. Statistical Analysis

Data was exported from the device software in comma-separated values format for analysis, and processed in a Microsoft Excel spreadsheet. The mean and median values of the most significant parameters, together with their standard deviations, are given in Table 4. 

Both sway path (length of the path followed by the CoP during the recording) and sway area (the area swept by the radius from the mean CoP to all the subsequent points of the path) have been “normalized” to the test recording time. The ratio between the sway area and the sway path is proportional to the postural control tone: when below normal, a hypertonic behavior is suspected; when above normal, a hypotonic behavior should be considered. Stay time, spatial distance and time distance are the sway density “structural” parameters: the spatial distance/stay time (SD/ST) ratio expresses the motor control efficiency; the lower the better. SPF is the count of “anomalies” with respect to the normal ranges.

**Figure 3:**
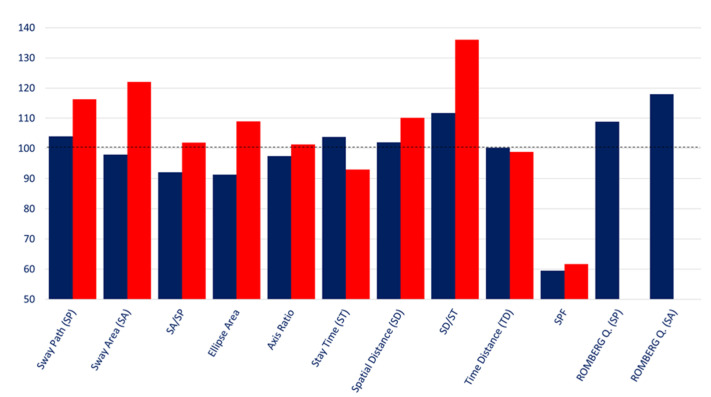
Cohort classical parameters (% of Normal Values).

The observed mean/median values of the classical parameters are almost identical to those of the healthy sample used as a comparison reference (see graph in Figure 3). 

Cohort mean values are close to normal. SPF was compared to the dysfunction threshold demonstrating the good mean condition of the cohort.

## 3. Results

The demographic and anthropometric data of the participants enrolled in the present study are shown in Table 2.

**Figure 4:**
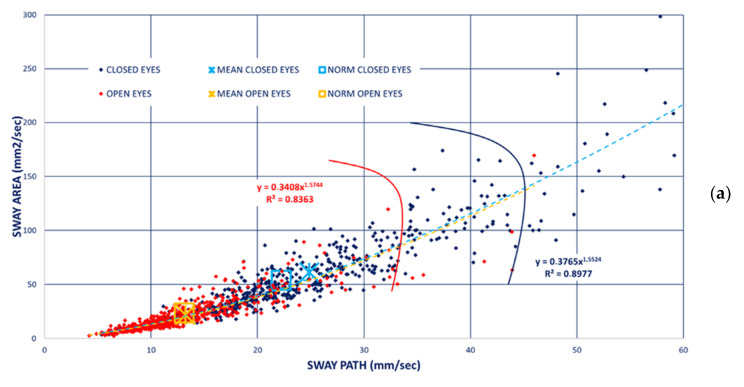

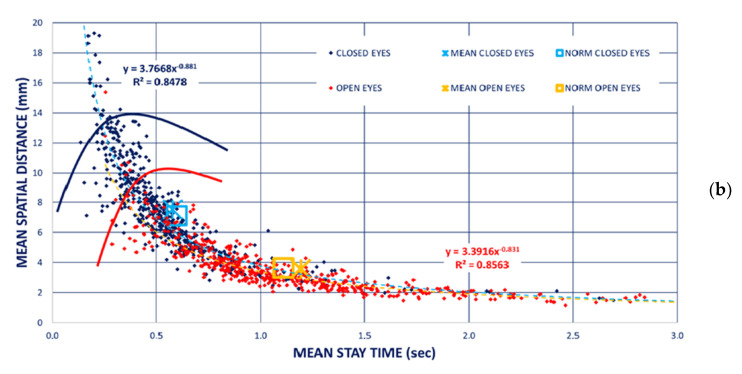
Cohort sway (**a**) and sway density (**b**) parameters.

Figure 4 shows the diffusion plots of the sway (sway area vs sway path) and sway density (mean spatial distance vs mean stay time): mean values are remarkably close to normal values. Both sway graphs show a strong correlation: *sway area* = 0.3408 × (*sway path*)^1.5524^ (open eyes) with a correlation R² = 0.8363; *sway area* = 0.3765 × (*sway path*)^1.5524^ (closed eyes) with a correlation R² = 0.8977; *mean spatial distance* = 3.3916 × (*mean stay time*)^−0.831^ (open eyes) with a correlation R² = 0.85693; *mean spatial distance* = 3.7668 × (*mean stay time*)^−0.881^ (closed eyes) with a correlation R² = 0.8478. Such high correlation values and the strong affinity between open and closed eyes may shed new light on the functions which contribute to balance control, at least in terms of suggesting a cybernetic “transfer function” warranting further study. 

Blue and Red arcs show tentative attention thresholds, respectively, for Closed and Open eyes tests for both Sway and Sway Density. Note the high R^2^ correlation values.

On the same plots, we have indicated arcs outlining the supposedly defective areas: it is difficult to give an exhaustive interpretation of what may simply be the effect of individual characteristics, both anthropometric and those related to physical attitude.

**Figure 5:**
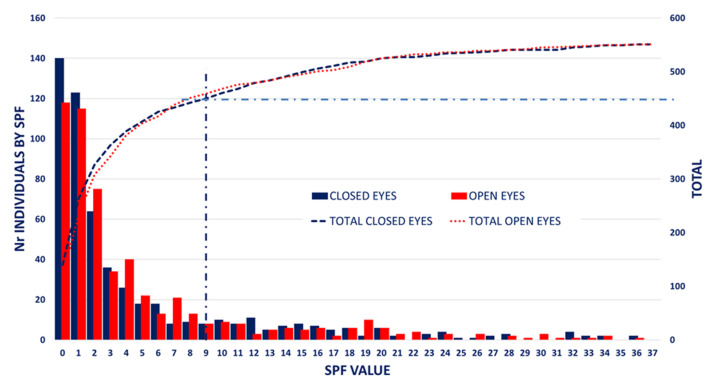
Cohort distribution by SPF. Please note that the two (Closed and Open Eyes) plotted values are almost superimposable.

Despite the close similarities to a “normofunctional” population, we found the SPF to be over the proposed attention threshold of 9 for 101 subjects (CE), 92 subjects (OE), and 48 subjects (both CE and OE). This means that out of a population of 551 subjects, we would recommend further diagnostic testing for 145 (30.1%) (Figure 5).

**Figure 6:**
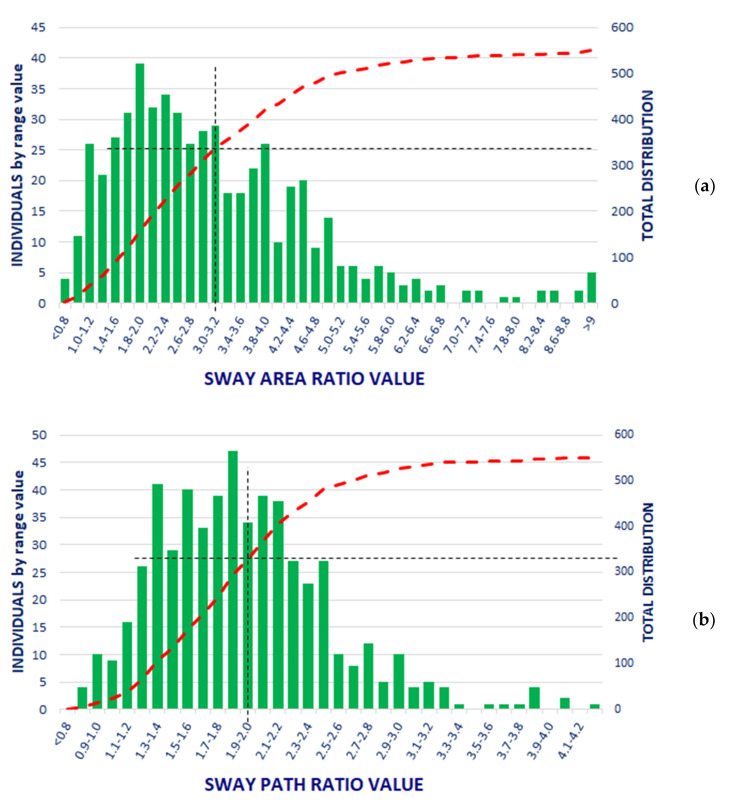
Distribution by Romberg’s ratio for sway area (**a**) and Sway Path (**b**).

Based on the proprioceptive deficit measured using Romberg’s quotients (sway path and sway area measured with closed vs open eyes), given an attention threshold of 2 (sway path) and 3 (sway area) (Figure 6), we would suggest further diagnostic testing for 220 subjects (50.7%).

Please note that almost 65% of the cohort shows values within normality range as indicated by the interrupted lines.

**Figure 7:**
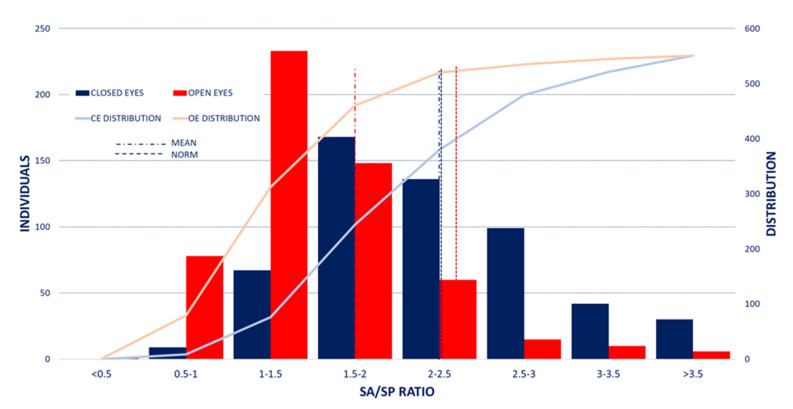
Cohort Postural Tone. The ratio between Sway Area and Sway Path is proportional to Postural Tone (under 1.5 a hypotonic behavior, whilst over 3 a hypertonic behavior will be observed). Although within normality range, a rather uncommon hypertonic condition in the Open Eyes distribution can be noticed for this cohort.

The postural tone provided additional information (Figure 7): in the closed eyes test, the cohort shows a mean value close to normal, while, in the open eyes test, there is a remarkable hypertonic trend, possibly attributable to fear of falling.

**Figure 8:**
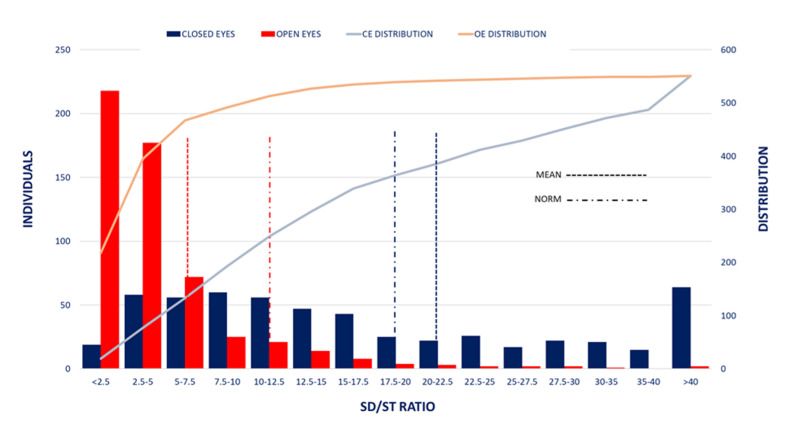
Cohort Motor Control Index. The ratio between the Mean Spatial Distance between subsequent temporary stabilization points and the Mean Stay Time on the same points is a measure of the performance of the motor control: the lower the better. The beneficial effect of Adapted Physical Activity (APA) practiced by the cohort is evident by comparison between mean and normality values in the open eyes test.

The motor control index obtained from the ratio between the mean spatial distance (the lower the better) and the mean stay time (the higher the better) should be as low as possible and is normally under 15.59 (closed eyes) and 4.56 (open eyes) (Figure 8).

The cohort of subjects, participants in an adapted physical activity program, showed good performances, close to the norm, in both open and closed eyes tests. A motor control deficit, however, was detected in 149 individuals within the normal test range, suggesting the need to modify their lifestyle with the addition of light physical activities. Ninety-one individuals with other types of dysfunctions (94) also showed a motor control deficit (97%). Only 9.3% of the subjects showed motor control performances warranting specific attention, which should not come as a surprise, given their participation in the APA courses: it should instead be seen as a confirmation of the APA program effectiveness.

**Figure 9:**
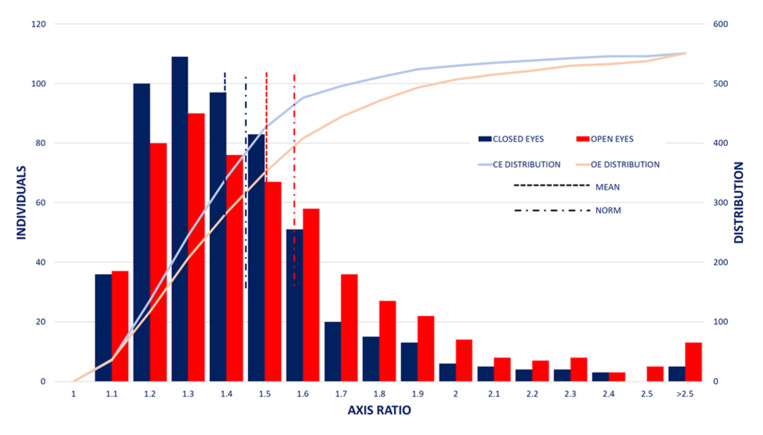
Ninety-five percent Confidence Ellipse Axes Ratio. The Ellipse Axes Ratio can give evidence of a preferential direction of the Sway whenever an evident unbalance is observed. Although the observed values are mostly within normal range, the red bars reporting the axis ratio in the Open Eyes test suggest the presence of some visual deficit for a few individuals of the cohort.

Analysis of the ellipse axis ratio (see Figure 9), which should not exceed the value of 2, suggested the following: 5 (0.8%) subjects with possible vestibular deficits (see Scheme 1) and 9 subjects with possible visual deficits (1.6%), and further analysis may permit a reduction in the ellipse axis ratio threshold.

**Scheme 2:**
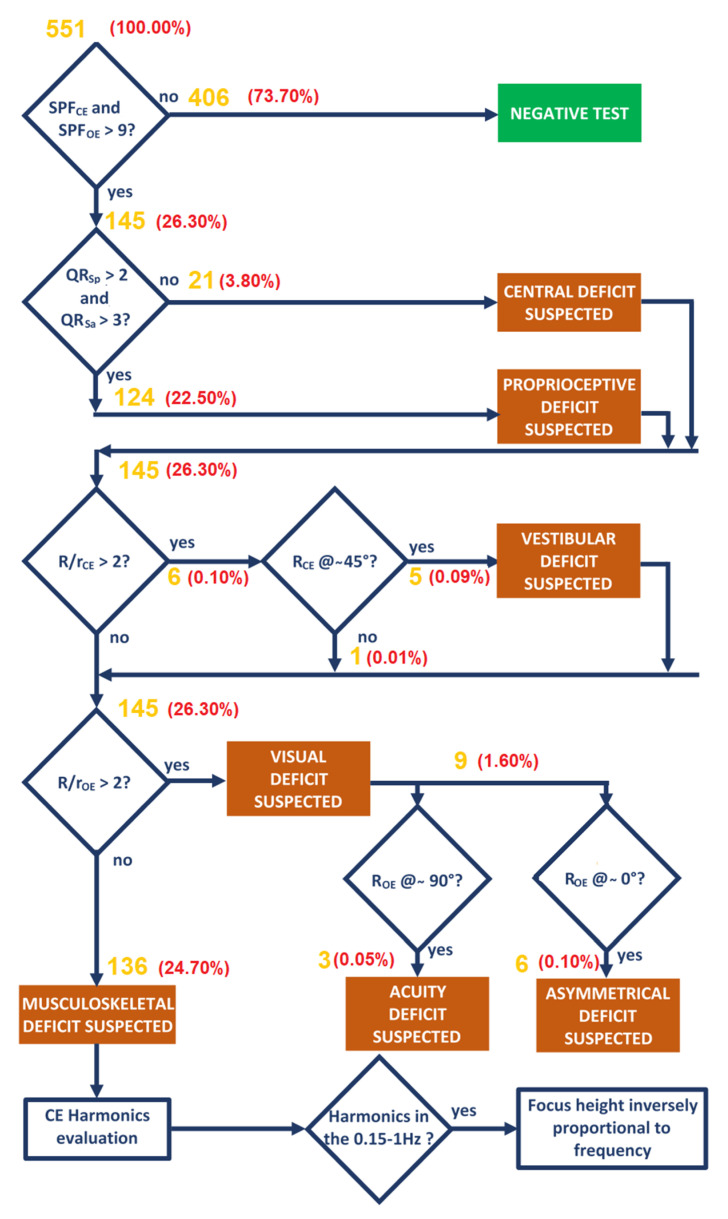
Outcome assessment for the cohort. This is the result of the automated assessment process as shown in Scheme 1 for the examined cohort.

Using the data analysis process described (Scheme 1) on a group of 551 compensated individuals aged over 65, we were able to recommend further specialist testing, as shown in Scheme 2.

## 4. Discussion

The findings of this study are consistent with our initial hypotheses. By screening an apparently “normofunctional” population using a non-invasive test requiring only basic computer skills to conduct, we identified a cluster of subjects at ROF for whom further diagnostic testing would be warranted. 

If this approach is confirmed by specific controlled studies, we would have identified the need for 145 specialist treatments out of a population of 551 individuals (26.3%) instead of over 2755 visits (neurological, neurovascular, vestibular, ophthalmological, physiatry/rheumatology) for the entire cohort. In addition to the inevitable resistance from the 406 subjects with no balance deficits, such an approach would constitute an unaffordable workload for the health institutions. The significant savings offered by the proposed approach, in terms of resources and related costs, are clear, appear to constitute a sustainable workload for the healthcare service, and would be an acceptable burden for the population.

The instrumented Romberg test has proven to be rapid and simple to perform: subjects were tested at an average interval of 12 min (5 patients/h), making this procedure suitable for use as a mass screening test. Given the intended use of the proposed balance assessment as a routine screening tool, the above semi-automatized process could result in an economically affordable process, capable of:

1. detecting dysfunctional subjects likely to be at greater ROF;
2. suggesting the most likely etiologies of the observed dysfunctions;
3. providing appropriate grounds for a prescription of further selective specialistic diagnostics (ENT, neurology, ophthalmology, osteopathy, chiropractor, physiotherapy, geriatrics, rheumatology) in an effort to reduce the ROF; and
4. producing an output clinical report that can be electronically forwarded to the patient’s caregiver for a comprehensive evaluation of their frailty and inclusion in their medical records.

Italian healthcare statistics report 1.57 million emergency room admittances annually for domestic fall-related injuries, data which presumably reflects the situation in the rest of Europe. Ten percent (157,000) of these injuries lead to hospitalization with an average stay of 8.4 days, at a daily cost of 3000 Euros [72]. In addition to the social benefit, a 10% reduction in these accidents would result in an estimated saving of 400 million Euros for the annual healthcare budget. To perform a yearly screening of the entire Italian population over the age of 65 (14 million persons) using an instrumented Romberg testing device capable of 8000 tests/year would require 1750 manned testing stations, at an estimated total annual cost of under 50 million Euros.

Healthcare 4.0 is an Italian developmental plan designed to increase the efficiency and effectiveness of the national health service, as is currently being done throughout Europe, largely based on telemedicine and digital data processing. The basic idea is to store each piece of test data in the cloud (labeled with a HASH code to protect patient sensitive data), which will be then securely processed using artificial intelligence (AI) algorithms [73].

The wide array of available numerical indicators will be processed using AI applications. Consider SPF with its 126 coordinates, applied to a subject whose patient history is expressed according to the ICD/ICF (International Code of Diseases/International Code of Functions) classification [74,75]. The main anthropometrics of the subject are also available. Each single recorded test parameter data set will be anamnestically labeled. By adding anamnestically labeled subjects to the database and inserting them in the 126-dimension virtual space defined by the 126 values, we will certainly observe a clustering of the subjects, and AI will gradually be able to suggest the most likely interpretation of the datasets.

In literature there is no consensus on the best tool to evaluate ROF in the elderly population. Some authors suggest that self-reporting measures in the form of questionnaires have demonstrated clinical utility as tools to identify fall risk [76], while the most widely used tools to identify ROF reported in previous studies include the Timed Up and Go test [77,78], the Timed 180° Turn test [79,80], the Tinetti test [81,82,83], the Functional Reach test [84], the Dynamic Gait Index [85], and the Berg balance scale [86,87].

In a systematic review and meta-analysis, Park et al. stated it would be preferable to have a high sensitivity test with low specificity, given that a good screening tool should be able to identify all the subjects at ROF to minimize this risk. On the other hand, to reduce the number of false positives and increase the diagnostic accuracy of the ROF, they conclude that two assessment tools used in combination would maximize the characteristics and predictability of each test [88]. However, these tests show a rather unexpected lack of technological approaches and would be unable to identify the likely etiology of the observed dysfunction or suggest a specialist diagnostic path to prevent unnecessary specialist treatment interventions.

Technological advances in sensors and processing appear to have supported bioengineering R&D (Research and Development) efforts, offering gait analysis systems, baropodometric analysis devices, mobile platforms, and accelerometric segment motion detectors, among others. Despite being extremely useful tools for detecting static and dynamic balance and gait characteristics, none of them are suitable for performing the instrumented Romberg test to accurately measure CoP position during unperturbed stance, nor for recommending further specialist treatment options.

This study has some limitations. First, the absence of any follow-up evidence to confirm the sensitivity/specificity of the proposed process. We suggest repeating the test in the five different areas, possibly in multicentric mode, administering the appropriate test in blind mode to subjects with specific deficits and to an equal number of subjects deemed to be free of such problems.

Another limitation of this study is that only female subjects have been included. This selection was made to guarantee a homogeneous cohort, since the initial sample resulted in 588 participants, of whom 551 were female and 37 were male. Although this limitation may potentially influence the final results, in literature, there is no consensus regarding gender influence on balance and postural stability. Hageman et al. reported no gender effect on postural control when functional scores of six analyzed balance parameters were normalized in relation to body height [89]. Moreover, the results of a study by Faraldo-Garcìa et al. showed that gender variables have no influence on the sensory contribution (visual, somatosensory and vestibular systems) to postural control [90].

A more comprehensive approach involving geriatrists could begin applying the method to better understand the complex relationship between balance and frailty, providing an insight into the question of whether balance deficit is an effect of frailty, or a factor which contributes to it.

## 5. Conclusions

Mass administration of the instrumented Romberg test using a force plate, while not capable of determining the risk of fall when used in isolation, may make a viable and valuable contribution to risk-of-fall assessment in tandem with other tools, and therefore to the timely, selective, and appropriate indication of further diagnostics for effective fall prevention, putting the lesson of Geoffrey Rose on a “population strategy” in healthcare into practice [91,92]. A number of studies have been conducted to measure the effects on balance-keeping performance using the proposed test following specific interventions [89,90], suggesting the additional value of the test in monitoring the evolution and worsening of performance.

## Figures and Tables

**Table 1:** Cohort anthropometrics. The cohort of females was subdivided in four groups, each with 5-year age range. Foot size is expressed using the EU system, where 35 corresponds to a foot length of 22.4 cm, and foot length increases by 0.66 cm with each size (45 = 29.0 cm). BMI (body mass index) was calculated according to the standard formula: BMI = kg/m^2^, where m is the subject height expressed in meters.

Parameter		Age 65–70		Age 71–75		Age 76–80		Age > 80	
		212 Subjects		159 Subjects		107 Subjects		73 Subjects	
		MEAN	STD DEV	MEAN	STD DEV	MEAN	STD DEV	MEAN	STD DEV
Height	Cm	157.58	5.92	158.07	6.33	157.05	5.60	156.15	2.55
Weight	Kg	65.43	11.83	66.81	11.03	63.95	10.09	63.79	11.54
Foot size	EU size	37.43	3.02	37.76	3.40	37.58	3.94	38.18	1.64
Age	Years	67.79	1.76	73.03	1.42	78.09	1.40	83.78	2.55
BMI	kg/m^2^	26.33	4.40	26.73	4.08	25.94	3.91	26.14	4.43

**Table 2:** Unperturbed upright stance: classical parameters.

Parameter	Description
Sway path	The length of the CoP path during the test
Mean CoP velocity	The ratio between the sway path and the recording time
Sway area	The area of the surface swept by the radius connecting the mean CoP to all subsequent path points
Normalized sway path	The value normalized to the recording time is equal to the mean CoP velocity
Normalized sway area	The value normalized to the recording time
Confidence ellipse area	The area of the smallest ellipse containing 95% of the points of the CoP path
Confidence ellipse axis	The major and minor axis of the confidence ellipse and the angle between the major axis and the antero-posterior sway axis
Harmonic analysis	The spectral distribution of the sway along the two axes (antero-posterior and latero-lateral)
Max oscillations	The maximum oscillation along the two axes (antero-posterior and latero-lateral)
Oscillations Std Dev	The standard deviations of the oscillations along the two axes (antero-posterior and latero-lateral)
Correlation index	The correlation among the oscillations along the two axes (antero-posterior and latero-lateral)

**Table 3:** Force plate characteristics. The device used in the test is the ArgoplusMK1^®^ designed and manufactured by Fremslife Srl (Genoa, Italy).

**Platform Size**	600 × 600 mm	**Weight Range**	15 to 200 kg
**Natural Frequency**	>300 Hz	**Type of Sensors**	Strain Gauges
**Output**	CoP Position (x,y) vs. time	**Sampling Rate**	100 Hz
**COP Accuracy**	<0.1 mm	**COP Resolution**	<0.09 mm
**Data Processing**	The CoP trajectory is low-pass filtered with a 2nd order Butterworth filter (cutoff frequency 12.5 Hz)		
**Computed Parameters**	Sway path, sway area, AP and ML oscillations, 95% confidence ellipse, harmonic analysis of x-y components in 8 bands, sway density, SPF, LOS		
**Display**	Stabilogram, statokinesiogram, spectral analysis		

**Table 4:** Main results: both sway path (length of the path followed by the center of pressure (CoP) during the recording) and sway area (the area swept by the radius from the mean CoP to all the subsequent points of the path) have been “normalized” to the test recording time. The ratio between the sway area and the sway path is proportional to the postural control tone: when below normal, a hypertonic behavior is suspected; when above normal, a hypotonic behavior should be considered. Stay time, spatial distance and time distance are the sway density “structural” parameters: the spatial distance/stay time (SD/ST) ratio expresses the motor control efficiency; the lower the better. SPF is the count of “anomalies” with respect to the normal ranges.

Parameter	Unit	Closed Eyes				Open Eyes			
		Mean	Std dev	Median	Norm	Mean	Std dev	Median	Norm
Sway path (SP)	mm/s	25.84	12.08	23.06	22.22	13.65	6.05	12.35	13.12
Sway area (SA)	mm^2^/s	65.71	54.12	51.56	53.84	23.27	17.88	18.18	23.75
SA/SP	mm	2.30	0.79	2.19	2.26	1.57	0.58	1.46	1.70
Ellipse area	mm^2^	420.82	286.66	358.34	386.37	194.69	143.93	152.12	213.26
Axis ratio	nr	1.39	0.32	1.33	1.38	1.48	0.36	1.40	1.52
Stay time (ST)	s	0.55	0.32	0.49	0.60	1.15	0.61	1.00	1.11
Spat. distance (SD)	mm	7.84	3.50	7.33	7.12	3.73	1.86	3.26	3.65
SD/ST	mm/s	21.21	19.96	15.04	15.59	5.10	6.26	3.35	4.56
Time distance (TD)	s	0.54	0.04	0.54	0.55	0.55	0.04	0.54	0.54
SPF	nr	5.55	7.65	2.00	<9	5.36	7.24	2.00	<9
Romberg Quotient (sway path)		1.94	0.58	1.86	1.78				
Romberg Quotient (sway area)		3.14	1.74	2.78	2.66				
